# Exploring the Link Between Social Capital and Edentulism in Low‐ and Middle‐Income Countries

**DOI:** 10.1002/cre2.70241

**Published:** 2025-10-14

**Authors:** Saida Ben‐Bihi, Yanfei Guo, Richard Olofsson, Hülya Çevik‐Aras, Junmei Miao Jonasson

**Affiliations:** ^1^ School of Public Health and Community Medicine, Institute of Medicine, Sahlgrenska Academy University of Gothenburg Gothenburg Sweden; ^2^ Shanghai Municipal Center for Disease Control and Prevention Shanghai China; ^3^ Specialist Clinic for Orofacial Medicine Uddevalla Public Dental Service Trollhättan Region Västra Götaland Sweden; ^4^ Department of Oral Medicine and Pathology, Institute of Odontology, Sahlgrenska Academy University of Gothenburg Gothenburg Sweden; ^5^ Section for Oral Biology and Immunopathology/Oral Medicine, Department of Odontology, Faculty of Health and Medical Sciences University of Copenhagen Denmark

**Keywords:** aging, edentulism, oral health, social capital

## Abstract

**Objectives:**

Although social capital (SC) has received growing attention in public health, no research has yet compared how SC and oral health are associated in diverse contexts. This cross‐sectional study aims to examine the association of community and individual‐level SC with edentulism among adults aged 50 and above in five low‐ and middle‐income countries (LMICs).

**Materials and Methods:**

Data are from SAGE‐wave 1 (2007–2010), including 27,676 participants aged 50 years or older from China (*N* = 12,830), Ghana (*N* = 4261), India (*N* = 6040), Mexico (*N* = 1327), and South Africa (*N* = 3218). The primary outcome was self‐reported edentulism. Cognitive and structural SC were used to measure SC at the individual and community levels. The association between SC dimensions and oral health was investigated using a two‐level multilevel logistic regression.

**Results:**

Multilevel analysis revealed that low individual and community structural SC are independently associated with edentulism in China (OR 1.43; CI 1.08–1.89 and OR 1.95; CI 1.32–2.90, respectively). In South Africa, low individual cognitive SC was negatively associated with edentulism (OR 0.48; CI 0.24–0.98), while low individual structural SC was positively associated with edentulism (OR 2.34; CI 1.16–4.74). In Ghana, only participants living in middle community structural SC had higher odds of being edentulous after adjustment for all potential confounders (OR 1.78; CI 0.71–2.96). In Mexico and India, no association was found between any dimensions of SC and oral health.

**Conclusion:**

The dimensions of SC and its relationship to edentulism differed across the five LMICs. Our study highlights the importance of considering contextual factors when analyzing the relationship between SC and oral health. Further research is required to understand how SC influences oral health in LMICs.

## 1 Introduction

Oral diseases are the most prevalent noncommunicable diseases globally, affecting approximately half the world's population. Among those affected, three out of four individuals live in low‐ and middle‐income countries (LMICs) (World Health Organization 2022). Despite the potential for cost‐effective interventions to prevent and manage oral diseases, oral health has long been neglected in the global health agenda (The Lancet 2009).

The burden of oral diseases in LMICs is substantial across age groups and socioeconomic strata, with sharp increases in untreated dental caries, periodontal disease, and tooth loss among older adults (Peres et al. 2019; FDI 2015). Globally, the population aged over 60 is projected to nearly double from 12% in 2015 to 22% by 2050, especially in urban areas (World Health Organization 2021). Yet, many LMICs face limited health resources and lack clear policies focused on the aging population (Huang et al. 2020). One often overlooked consequence is edentulism, which affects nutrition, quality of life, and social inclusion among older individuals (Gil‐Montoya et al. 2015). In addition, high out‐of‐pocket costs for oral care impose heavy financial burdens on individuals and families (World Health Organization 2022; Peres et al. 2019).

Oral health disparities are closely tied to broader social and structural determinants of health (FDI 2015; Singh et al. 2018; World Health Organization 2010). Social capital (SC), defined by Putnam as “features of social organisation such as trust, norms and networks that can improve the efficiency of the society by facilitating coordinated actions” (Putnam 2000)—has gained increasing recognition as a critical determinant of health and health equity (Kawachi and Berkman 2000; Ehsan et al. 2019; Carlson and Chamberlain 2003). Numerous studies have documented positive associations between SC and overall health, including better self‐rated health, improved mental well‐being, and lower mortality (Hyyppä 2010; Nieminen et al. 2013; Holt‐Lunstad et al. 2010; Gilbert et al. 2013; Murayama et al. 2012; De Silva et al. 2005). Despite increasing recognition of SC's role in health, its association with oral health—particularly in LMICs—remains understudied, representing a key gap this study aims to address. A recent cross‐sectional study indicates that living in a community with low SC and being a person with low SC increases edentulism risk among Chinese individuals aged 50 and older (Li et al. 2022). A longitudinal cohort study conducted in Japan has shown that older adults with less social support or participation are more likely to have fewer teeth (Koyama et al. 2016).

There is limited research from LMICs examining how SC is conceptualized and how it relates to oral health outcomes. This lack of contextual evidence poses a challenge to effectively expanding the potential benefits of SC to improve health in underrepresented settings (Han et al. 2012). While global frameworks encourage addressing social determinants of health, assuming uniformity in their effects across contexts may be misleading. Evidence suggests that the relevance and strength of SC may vary by region, shaped by historical, economic, and cultural differences. For example, in China, the potential health effects of SC may differ between northern and southern regions, which exhibit distinct development trajectories and social traditions (Yuan et al. 2022). According to Story (2013), macrolevel environmental risk factors such as economic inequality or low community trust vary significantly across countries, potentially shaping how SC influences health outcomes.

Considering these complexities, a multicountry approach can help explore context‐specific mechanisms and provide comparative insights. LMICs differ in oral health awareness, cultural norms around aging and dental care, and family structures. Urbanization, migration, and weakening family support systems further complicate elder oral health, particularly in rural regions. Understanding the interaction between SC and oral health in LMICs is thus critical to inform locally relevant public health strategies, resource allocation, and future interventions.

Therefore, the current study aims to investigate the association of different dimensions of SC with oral health among adults in five LMICs (China, Ghana, India, Mexico, and South Africa).

## 2 Methods

### 2.1 Study Setting

The present study employed a quantitative cross‐sectional design, utilizing WHO SAGE Wave 1 data, which was retrieved from the publicly accessible WHO SAGE data repository. This longitudinal survey was conducted across six developing nations (China, Ghana, India, Mexico, Russian Federation, and South Africa between 2007 and 2010; only five countries were included in the current study (China, Ghana, India, Mexico, and South Africa). These countries collectively encompass a substantial portion of the global population and exhibit diverse geographic and socioeconomic contexts. The WHO‐SAGE employed a stratified random sampling strategy to achieve nationally representative samples at the household level.

### 2.2 Study Population

Populations were stratified based on factors such as urban versus rural location, socioeconomic status, and regional characteristics. Random samples were then drawn from each stratum to ensure proportional representation across all subgroups.

The WHO SAGE survey included two distinct age cohorts: individuals aged 50 years and older and a smaller group aged 18–49. For the current analysis, only participants aged 50 years and above were included. Individuals with missing responses on the primary outcome variable (edentulism) were also excluded to ensure data completeness.

The raw data were cleaned, recoded, and prepared for analysis using Stata SE 17 software (Stata Corp LLC). The final analytical sample comprised 27,676 adults aged 50 and above across five countries: China (*N* = 12,830), Ghana (*N* = 4261), India (*N* = 6040), Mexico (*N* = 1327), and South Africa (*N* = 3218).

Missingness in other covariates was minimal (< 15%) and was addressed as outlined in Section 2.5.

### 2.3 Data Collection

Data collection was conducted by trained interviewers from national statistics offices, universities, and local organizations in each country, under the guidance of the World Health Organization (WHO). Face‐to‐face interviews were utilized to gather data from individuals on sociodemographic factors, health conditions, health care utilization, quality of life, social cohesion, tooth loss, and difficulties with the respondent's mouth or teeth. The questionnaire was first developed and tested in English and translated into local languages to ensure cultural and linguistic relevance. In addition, a pilot study in three developing countries was conducted to enhance validity.

For the purposes of this study, only questions relevant to SC and oral health were included in the analysis. The SAGE generic questions are available as external resources through the WHO data archive. Survey response rates by country are as follows: China (2008–10; 93% received), Ghana (2008–09; 80% received), India (2007–08; 68% received), Mexico (2009–10; 51% received), and South Africa (2007–08; 77% received).

### 2.4 Measures

#### 2.4.1 Dependent Variable

The primary outcome variable was edentulism, defined as the complete absence of all natural teeth. Participants were asked, “Have you lost all of your natural teeth?” Responses were coded as 1 for “yes” (edentulous) and 0 for “no” (nonedentulous). While self‐reported edentulism is commonly used in large‐scale epidemiological studies and has shown reasonable validity compared with clinical assessments, it may be subject to reporting or recall bias. This limitation is acknowledged and further discussed in the discussion section.

#### 2.4.2 Independent Variables

This study utilized a conceptual framework, considering the cognitive and structural dimensions of SC at the individual and community levels, to examine the relationship between SC and oral health. Measuring SC lacks a standard method and necessitates indicators. Previous studies on Chinese and Japanese adults (Li et al. 2022; Koyama et al. 2016) used a similar approach to assess different dimensions of SC at various levels, as outlined below:

At the *individual level*, cognitive SC is measured through perceived community trust and safety, based on a total of five survey questions. Three questions assessed perceived community trust by asking respondents to rate trust in neighbors, coworkers, and strangers on a 5‐item Likert scale (1 = very great extent to 5 = very small extent). Responses were reversed, with coding ranging from 1 = very small extent to 5 = very great extent.

For perceived community safety, participants responded to two questions: (1) “How safe do you feel when alone at home from crime and violence?” and (2) “How safe do you feel walking down your street alone after dark?” Ratings used a 5‐item Likert scale (1 = completely safe to 5 = not safe at all). Responses were reversed and coded as 1 = not safe at all to 5 = completely safe. The final individual cognitive SC score was computed by summing perceived community trust and safety scores and then categorized into tertiles (high, middle, low). Higher scores indicate elevated individual cognitive SC. Li et al. conducted a cross‐sectional study among Chinese adults and reported an acceptable Cronbach's *α* of 0.6638, demonstrating reliability (Hyyppä 2010).

At the individual level, structural SC involves forming social networks through actual behavior. A previous study by Zamora‐Macorra et al. used a social cohesion index with nine questions to measure community engagement over the past year, including religious services, clubs, groups, and unions (Zamora‐Macorra et al. 2017). Our study applied the same approach to assess structural SC, examining formal and informal social activity frequency. Nine survey questions covered: (1) public meetings attendance, (2) religious services participation, (3) social gatherings, visiting friends/relatives, (4) groups, clubs, unions involvement, (5) meeting community leaders, (6) interacting with neighbors, (7) hosting friends, (8) visiting different neighborhood homes, and (9) coworker socializing. Responses ranged from 1 to 5 (never = 1, every day = 5), with scores summed and divided into tertiles with higher scores indicating greater perceived cohesion. A previous study conducted on Chinese adults aged 50 and older, using a similar method, reported a Cronbach's *α* of 0.6246, indicating an acceptable measure (Li et al. 2022). We recognize that the moderate Cronbach's *α* values observed (0.62–0.66) reflect the multidimensional and complex nature of SC constructs. Such values are consistent with prior large‐scale studies and are considered acceptable for examining associations with health outcomes, including oral health.

**Figure 1:**
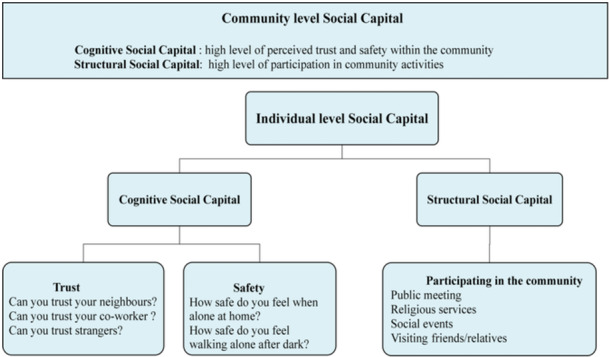
Components of social capital.

At the *community level*, individual cognitive SC scores were aggregated, and the mean score for each community was calculated to derive community‐level cognitive SC. In this study, “community” refers to the primary sampling units (PSUs) defined in the WHO SAGE survey, which typically correspond to geographically bounded areas, such as villages or urban neighborhoods. This method is consistent with the survey's multistage sampling framework and facilitates meaningful aggregation of contextual‐level indicators. Communities were then categorized into tertiles—low, medium, and high—based on the distribution of mean scores. This categorization approach, widely used in public health research (Li et al. 2022; Aida et al. 2009; Santiago et al. 2013), simplifies interpretation, allows for comparison across groups, and is appropriate given the large, diverse sample. While categorization may lead to some loss of precision, it was selected to enhance interpretability and model stability across countries with differing SC profiles. Similar steps were taken for deriving structural SC measurement at the community level. Figure 1 illustrates the components of SC evaluated in this study.

#### 2.4.3 Covariates

The variables included in the study were chosen for their potential relevance to oral health based on relevant research literature (Kailembo et al. 2016).

Five *sociodemographic factors* were included: sex, age, residence, education, and wealth status. Sex was categorized as male or female. Age was continuous and grouped into 50–59, 60–69, and 70+ years. Residence was urban or rural. Marital status ranged from never married to widowed and was recategorized as married/cohabiting or not. Education levels included no schooling, primary/less, secondary, high school, and over. All variables were derived from the same individual questionnaires. SAGE provided household income as a variable in the data set, precategorized into five income quintiles—quintile 1 as poorest and quintile 5 as richest (Ferguson et al. 2003).

##### 2.4.3.1 Health Behaviors

Smoking status was measured by asking the first question, “Have you ever smoked or used smokeless tobacco?” Those who said “yes” were asked, “Do you currently use (smoke, sniff, or chew) any tobacco products, such as cigarettes, cigars, pipes, chewing tobacco, or snuff?” Respondents were divided into groups (nonsmoker, daily smoker, nondaily smoker, and former smoker). Alcohol consumption was categorized into nondrinker or drinker (nonfrequent, frequent, and heavy drinker). Nutritional status was determined from responses to the question, “How many portions of fruit do you eat daily?” and “How many vegetable servings do you consume every day?” Insufficient fruit and vegetable consumption was defined as less than five servings of fruits and/or vegetables per day (World Health Organization 2003).

##### 2.4.3.2 Chronic Diseases

Eight common chronic diseases (arthritis, diabetes, angina, stroke, chronic lung diseases, asthma, depression, and hypertension) were assessed through self‐report. Respondents reply to the question, “Have you ever been diagnosed with the above chronic condition?” with “yes” or “no.” The total number of chronic diseases was determined and grouped into “no chronic diseases or chronic diseases (if participants have one chronic disease, two chronic diseases, three to eight chronic diseases).

### 2.5 Statistical Analysis

Data were analyzed using Stata SE17 software (Stata Corp. Inc.). Participants under the age of 50 were excluded from the analysis. To account for the complex, multistage sampling design of the SAGE survey, all analyses incorporated survey weights, stratification, and clustering variables specific to each country. Normalized individual weights were applied to ensure nationally representative estimates. PSUs and stratification codes were used within Stata's “svy” command framework to adjust for design effects and enabling valid cross‐country comparisons. Missing data on covariates were minimal (< 15%) and were ignored in the analysis due to their limited extent and negligible impact on the results. The assessment of multicollinearity through the variance inflation factor (VIF) demonstrated its absence (with a mean VIF of 1.17), thereby enabling the incorporation of all variables in the multiple logistic regression analysis. A multilevel logistic model with individuals (first level) and communities (second level) was used to assess the association between SC dimensions and edentulism. For each country, the following models were employed:

- Model 0: A baseline model to compare edentulism variations across communities, assessing whether multilevel models were necessary. The intraclass correlation coefficients (ICCs) showed that a meaningful portion of the variation in edentulism was due to community‐level differences: China (13%), Ghana (20%), South Africa (64%), India (13%), and Mexico (35%). These results support the use of multilevel modeling in the analysis (Table 5).
- Model 1: It evaluates the impact of cognitive and structural SC variables on edentulism at the community level.
- Model 2: Individual SC variables were added to Model 1 to adjust for individual‐level effects.
- Model 3: Building on Model 2, individual and community SC variables were incorporated, along with sociodemographic factors (sex, age, marital status, residence, education, wealth quintile) to account for potential confounding.
- Model 4 included all variables; behavioral risk factors (smoking, alcohol use, nutritional status) and chronic diseases were added to Model 3.

**Figure 2:**
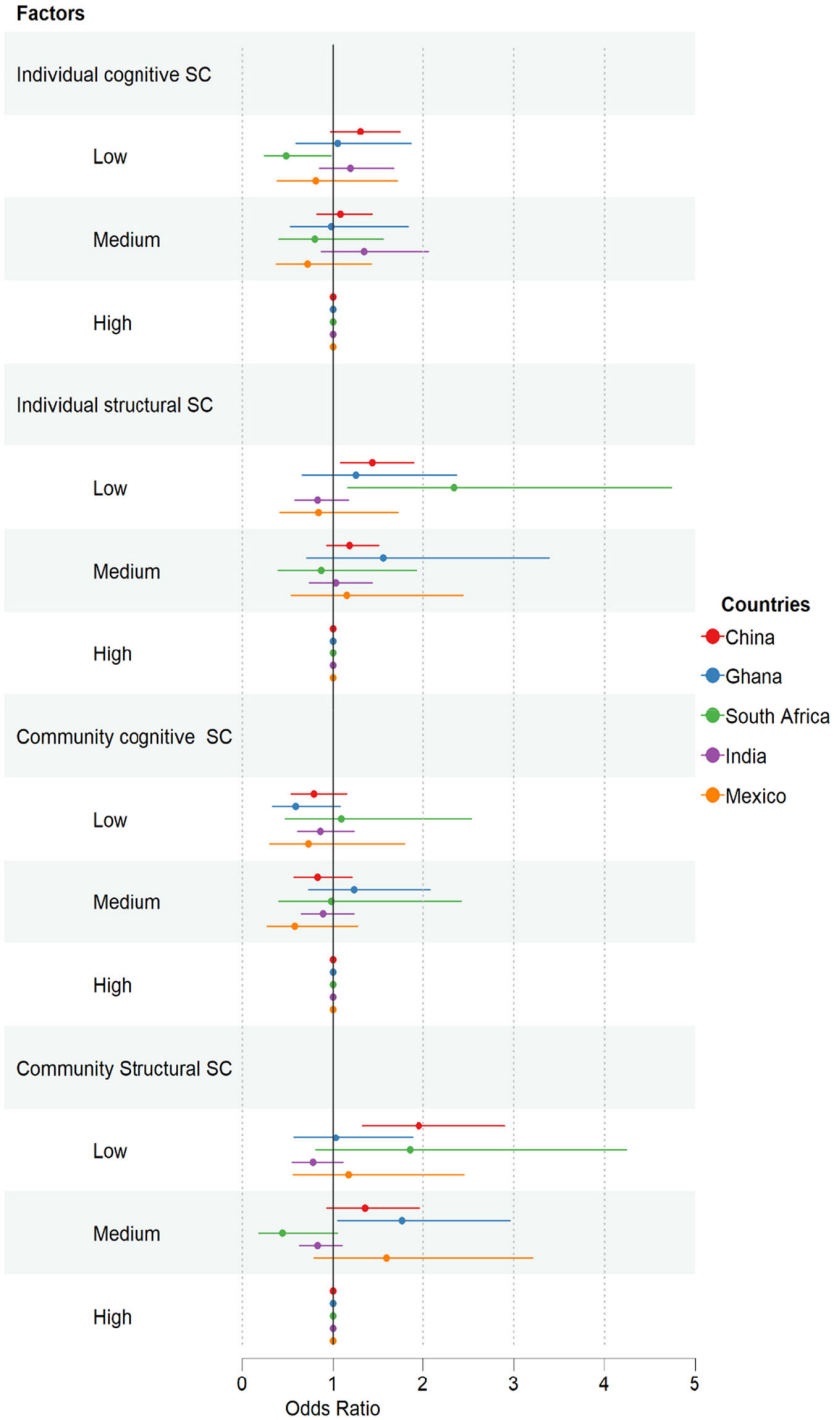
Forest plot illustrating associations between social capital and edentulism through multilevel logistic regression (Model 4). The *y*‐axis displays the explanatory variables influencing edentulism, while the *x*‐axis shows the adjusted odds ratios (aOR), with high social capital as the reference. SC, social capital.

The ICC for each model was calculated, reflecting the proportion of variance attributed to the community level. Regression results are displayed as odds ratios (ORs) with 95% CIs and a significance level of *p* < 0.05. RStudio was used to create a forest plot (Figure 2) to visually display the results for multilevel analysis (Model 4).

## 3 Results

The final analytical sample comprised 27,676 individuals aged 50 years or older from five LMICs: China (*n* = 12,830), India (*n* = 6040), Ghana (*n* = 4261), South Africa (*n* = 3218), and Mexico (*n* = 1327). Mean ages ranged from 61.6 years in India to 64.2 years in Ghana, with standard deviations of 8.9 and 10.6, respectively.

Edentulism prevalence varied across countries: China (11%; 95% CI: 10–11.5), Ghana (10%; 95% CI: 9.0–11.1), India (14.1%; 95% CI: 13.2–15.0), Mexico (24.7%; 95% CI: 22.4–27.1), and South Africa (10%; 95% CI: 9.0–11). Table 1 summarizes descriptive statistics of sociodemographic and health‐related variables by country. Table 2 presents edentulism distribution, indicating higher rates among older adults (70+ ), women (except in South Africa), and those unmarried or residing in rural areas, with some country‐specific variations.

**Table 1:** Weighted sample characteristics of adults aged 50+ years by country (*N* = 27,676).

	China	Ghana	India	Mexico	South Africa
Characteristics	*N* = 12,830	*N* = 4261	*N* = 6040	*N* = 1327	*N* = 3218
	*n* (%)	*n* (%)	*n* (%)	*n* (%)	*n* (%)
Sex					
Male	6013 (49.8)	2226 (52.3)	3179 (52.4)	630 (57.2)	1382 (45.3)
Female	6800 (50.2)	2035 (47.7)	2861 (47.6)	697 (42.8)	1834 (54.7)
Age group					
50–59	5596 (45.2)	1678 (39.9)	2774 (49.3)	312 (53.7)	1450 (50.8)
60–69	3842 (31.9)	1195 (27.6)	2032 (30.6)	551 (25.1)	1022 (30.3)
70+	3375 (22.8)	1388 (32.5)	1234 (20.1)	464 (21.3)	744 (19.0)
Marital status					
Married/cohabiting	10,672 (85.3)	2408 (58.9)	4533 (77.5)	840 (72.6)	1687 (55.7)
Unmarried/noncohabiting	2140 (14.7)	1853 (41.1)	1507 (22.5)	487 (27.4)	1529 (44.3)
Residence					
Urban	6304 (47.7)	1734 (40.8)	1510 (28.9)	970 (78.9)	2126 (64.2)
Rural	6509 (52.3)	2527 (59.2)	4530 (71.1)	357 (21.1)	1089 (35.8)
Wealth quintile					
Q1 (lowest)	2541 (16.1)	854 (18.4)	1020 (18.6)	287 (13.9)	613 (20.7)
Q2	2521 (18.1)	843 (19.2)	1131 (19.6)	286 (26.4)	649 (19.7)
Q3	2572 (20.4)	848 (20.5)	1112 (18.9)	228 (14.3)	632 (18.3)
Q4	2628 (23.5)	862 (20.6)	1286 (19.5)	269 (17.6)	655 (20.0)
Q5 (highest)	2509 (21.9)	849 (21.3)	1453 (23.4)	255 (27.8)	655 (21.2)
Education					
No schooling	3112 (22.3)	2329 (53.9)	3018 (50.6)	231 (12.3)	720 (24.1)
Primary or less	4874 (40.3)	886 (21.3)	1558 (24.9)	848 (64.9)	1287 (46.2)
Secondary	2578 (20.1)	172 (4.0)	624 (10.3)	105 (10.1)	371 (14.6)
High school and over	2249 (17.3)	847 (20.8)	840 (14.2)	143 (12.7)	330 (15.2)
Smoking					
Nonsmoker	8527 (64.1)	3150 (74.9)	2748 (44.1)	793 (55.5)	2104 (67.6)
Former smoker	791 (6.6)	577 (14.3)	321 (4.7)	235 (19.2)	284 (9.5)
Nondaily smoker	317 (2.5)	116 (2.6)	180 (2.9)	98 (7.9)	128 (3.4)
Daily smoker	3124 (26.8)	416 (8.1)	2790 (48.4)	201 (17.5)	695 (19.5)
Alcohol use					
Nondrinker	8830 (66.0)	1761 (42.0)	5054 (83.9)	640 (37.6)	2365 (75.8)
Drinker	3947 (34.0)	2496 (58.0)	986 (16.1)	687 (62.4)	830 (24.2)
Nutritional status					
Insufficient	2556 (16.8)	3408 (78.7)	5696 (95.2)	1193(90.9)	2662 (78.5)
Sufficient	9378 (83.2)	853 (21.3)	344 (4.8)	134 (9.1)	554 (21.5)
Health status					
No chronic diseases	6168 (50.0)	2942 (68.2)	3485 (58.0)	574 (52.7)	1605 (50.8)
Chronic diseases	6371 (50.0)	1314 (31.8)	2553 (42.0)	2861 (47.6)	1604 (49.2)

**Table 2:** Distribution of edentulism within each group of covariables and within each dimension of social capital in the five LMICs (weighted %).

Characteristics	China (*N* = 12,830)	Ghana (*N* = 4261)	India (*N* = 6040)	Mexico (*N* = 1327)	South Africa (*N* = 3218)
Sex					
Male	512 (8.1)	58 (2.6)	438 (13.8)	137 (18.0)	121 (8.3)
Female	643 (10.1)	68 (3.3)	470 (16.3)	155 (27.3)	139 (7.9)
Age group					
50–59	162 (2.8)	30 (1.7)	284 (9.5)	103 (14.5)	98 (6.0)
60–69	344 (8.5)	29 (2.4)	273 (14.8)	59 (17.9)	77 (7.9)
70+	648 (22.3)	68 (4.9)	350 (28.9)	129 (45.8)	84 (13.7)
Marital status					
Married/cohabiting	828 (7.6)	56 (2.2)	611 (13.1)	165 (17.1)	149 (8.4)
Unmarried/noncohabiting	327 (17.5)	69 (4.0)	297 (21.9)	127 (34.9)	109 (7.7)
Residence					
Urban	463 (7.6)	70 (4.0)	321 (18.4)	200 (19.1)	225 (10.9)
Rural	693 (10.4)	55 (2.2)	587 (13.7)	92 (32.8)	34 (3.0)
Wealth quintile					
Q1 (lowest)	315 (15.5)	22 (2.8)	178 (15.9)	45 (24.8)	21 (3.3)
Q2	247 (10.8)	21 (2.6)	162 (13.7)	148 (42.2)	630 (4.5)
Q3	265 (10.2)	28 (3.2)	156 (13.7)	35 (18.8)	587 (6.5)
Q4	191 (6.5)	23 (2.7)	191 (16.3)	38 (16.2)	641 (9.4)
Q5 (highest)	137 (4.9)	30 (3.4)	218 (15.5)	25 (6.8)	680 (16.3)
Education					
No schooling	503 (17.7)	76 (3.3)	493 (16.1)	46 (28.3)	18 (2.9)
Primary or less	453 (8.8)	22 (2.4)	236 (15.7)	215 (25.0)	113 (9.5)
Secondary	127 (4.9)	4 (2.2)	66 (10.7)	9 (7.0)	55 (14.7)
High school and over	73 (3.3)	24 (2.7)	112 (13.1)	21 (12.3)	29 (7.6)
Smoking					
Never smoker	746 (9.2)	92 (2.9)	422 (15.9)	213 (29.0)	146 (6.7)
Former smoker	115 (13.7)	24 (3.9)	55 (19.8)	31 (12.3)	40 (13.2)
Nondaily smoker	18 (5.5)	1 (0.6)	32 (18.2)	15 (14.2)	2 (1.9)
Daily smoker	268 (7.9)	9 (2.6)	398 (13.6)	33 (14.1)	71 (11.4)
Alcohol use					
Never drinker	815 (9.7)	58 (3.2)	806 (15.9)	152 (30.5)	207 (8.6)
Drinker	336 (7.8)	68 (2.7)	102 (10.5)	140 (16.9)	49 (6.4)
Nutritional status					
Insufficient	293 (14.4)	96 (2.9)	855 (14.9)	267 (22.2)	220 (8.7)
Sufficient	794 (7.9)	29 (3.3)	53 (18.3)	25 (20.4)	39 (5.7)
Health status					
No chronic diseases	438 (7.0)	66 (2.3)	460 (13.1)	137 (19.6)	74 (4.5)
Chronic diseases	679 (10.9)	60 (4.0)	447 (17.7)	155 (24.6)	183 (11.6)
Individual cognitive SC					
Low	448 (10.4)	52 (3.2)	292 (14.4)	105 (21.2)	55 (5.4)
Middle	444 (8.6)	33 (2.6)	356 (17.2)	63 (18.2)	80 (7.6)
High	264 (8.1)	40 (3.0)	259 (13.4)	123 (25.6)	123 (10.9)
Individual structural SC					
Low	552 (12.9)	48 (3.3)	351 (15.8)	133 (28.5)	144 (13.0)
Middle	347 (8.4)	51 (3.2)	316 (14.9)	90 (22.1)	68 (6.4)
High	256 (6.0)	26 (2.1)	241 (14.2)	68 (15.2)	46 (4.5)
Community cognitive SC					
Low	402 (9.0)	35 (2.2)	301 (13.6)	159 (24.7)	67 (6.9)
Middle	346 (9.4)	57 (4.1)	237 (14.2)	68 (19.5)	49 (5.1)
High	408 (8.9)	33 (2.6)	370 (17.2)	65 (19.5)	143 (11.1)
Community structural SC					
Low	349 (11.4)	30 (2.2)	332 (14.5)	85 (19.9)	152 (15.4)
Middle	400 (8.6)	66 (4.5)	265 (13.4)	121 (27.5)	50 (4.7)
High	406 (8.1)	29 (2.1)	311 (17.6)	86 (18.7)	56 (4.9)

Univariate logistic regression analyses (Table 3) revealed that age ≥ 70 was consistently associated with edentulism. In China and Mexico, edentulism was linked to female gender, low education, unmarried status, low income, alcohol use, chronic disease, and certain health behaviors. In South Africa, higher edentulism odds were observed among individuals with secondary education, rural residence, alcohol use, and chronic disease. In India and Ghana, associations were primarily seen with marital status and chronic conditions. Educational status showed no significant association with edentulism in Ghana.

**Table 3:** Association of dimensions of social capital and covariates with edentulism by country (crude odds ratio (95% CI).

	China	Ghana	India	Mexico	South Africa
Sex					
Male	reference	reference	reference	reference	reference
Female	1.30 (1.15–1.47)***	1.37 (0.89–2.12)	1.18 (0.86–1.63)	2.22 (1.34–3.70)**	0.98 (0.52–1.81)
Age group					
50–59	reference	reference	reference	reference	reference
60–69	3.37 (2.71–4.19)***	1.30 (0.67–2.54)	1.86 (1.45–2.38)***	2.44 (1.19–4.99)**	1.71 (0.96–3.04)
70+	12.09 (9.32–15.69)***	2.71 (1.58–4.65)***	4.96 (3.72–6.62)***	9.83 (4.53–21.36)***	2.54 (1.39–4.64)**
Marital status					
Married/cohabiting	reference	reference	reference	reference	reference
Unmarried/noncohabiting	2.60 (2.20–3.07)***	1.80 (1.13–2.86)*	1.85 (1.45–2.37)***	2.84 (1.69–4.78)***	1.05 (0.52–2.14)
Residence					
Urban	reference	reference	reference	reference	reference
Rural	1.49 (1.12–1.97)**	0.53 (0.34–0.82)**	0.85 (0.59–1.22)	1.35 (0.64–2.85)	0.30 (0.11–0.82)*
Wealth quintile					
Q1 (lowest)	3.73 (2.72–5.12)***	0.92 (0.43–1.98)	1.06 (0.75–1.49)	5.13 (2.18– 12.09)***	0.36 (0.11–1.19)
Q2	2.13 (1.50–3.01)***	0.86 (0.42–1.73)	0.91 (0.63–1.32)	5.06 (2.35–10.89)***	0.57 (0.17–1.93)
Q3	1.91 (1.40–2.59)***	0.98 (0.44–2.19)	0.84 (0.61–1.16)	2.49 (1.18–5.22)**	0.50 (0.22–1.18)
Q4	1.28 (0.92–1.78)	0.83 (0.42–1.63)	1.06 (0.70–1.60)	2.32 (1.07–5.05)*	0.75 (0.33–1.72)
Q5 (highest)	reference	reference	reference	reference	reference
Education					
No schooling	7.00 (4.84–10.12)***	1.35 (0.75–2.41)	1.42 (1.01–2.00)*	4.61 (1.70–12.45)**	0.65 (0.23–1.80)
Primary or less	2.89 (2.00–4.17)***	0.97 (0.38–2.48)	1.41 (0.98–2.02)	2.32 (0.94–5.70)	1.31 (0.93–3.74)
Secondary	1.51 (1.04–2.18)*	0.92 (0.31–2.78)	0.86 (0.53–1.37)	0.18 (0.15–1.91)	3.29 (1.33–8.15)*
High school and over	reference	reference	reference	reference	reference
Smoking					
Never smoker	reference	reference	reference	reference	reference
Former smoker	1.60 (1.24–2.06)***	1.26 (0.56– 2.80)	1.35 (0.75–2.43)	0.37 (0.18–0.77)**	1.59 (0.60–4.18)
Nondaily smoker	0.59 (0.36–0.96)*	0.22 (0.03–1.58)	1.30 (0.71–2.38)	0.66 (0.23– 1.95)***	0.32 (0.07–1.56)
Daily smoker	0.79 (0.66–0.95)*	0.95 (0.48–1.89)	0.93 (0.74–1.17)	0.36 (0.06–2.31)	0.87 (0.47–1.64)
Alcohol use					
Never drinker	reference	reference	reference	reference	reference
Drinker	0.76 (0.66–0.87)***	0.85 (0.58–1.24)	0.72 (0.52–1.00)	0.28 (0.14– 0.56)***	0.29 (0.13– 0.64)**
Nutritional status					
Insufficient	1.83 (1.52–2.20)***	0.98 (0.55–1.76)	0.90 (0.46– 1.77)	1.22 (0.47–3.15)	1.65 (0.69–3.96)
Sufficient	reference	reference	reference	reference	reference
Health status					
No chronic diseases	reference	reference	reference	reference	reference
Chronic diseases	1.66 (1.40–1.97)***	1.83 (1.20–2.79)**	1.44 (1.17– 1.77)***	2.40 (1.02–5.67)*	2.20 (1.16–4.16)*
Individual cognitive SC					
Low	1.35 (1.04– 1.75)**	1.07 (0.58–1.99)	1.31 (0.95–1.80)	0.78 (0.38–1.60)	0.55 (0.28–1.09)
Middle	1.08 (0.82–1.41)	0.91 (0.48–1.69)	1.46 (0.98–2.38)	0.71 (0.35–1.44)	0.65 (0.34–1.27)
High	reference	reference	reference	reference	reference
Individual structural SC					
Low	2.42 (1.90–3.09)***	1.70 (0.99–2.91)	1.23 (0.89–1.68)	1.06 (0.51–2.19)	2.58 (1.32–5.05)**
Middle	1.48 (1.20–1.83)***	1.69 (0.88–3.27)	1.15 (0.86–1.55)	2.00 (0.66–6.01)	1.35 (0.69–2.62)
High	reference	reference	reference	reference	reference
Community cognitive SC					
Low	0.98 (0.69–1.39)	0.84 (0.49–1.45)	0.97 (0.73–1.29)	0.58 (0.27–1.25)	0.65 (0.33–1.27)
Middle	1.15 (0.78–1.68)	1.49 (0.89–2.50)	0.89 (0.68–1.18)	0.64 (0.30–1.33)	0.47 (0.20–1.08)
High	reference	reference	reference	reference	reference
Community structural SC					
Low	1.74 (1.21–2.51)***	1.01 (0.57–1.78)	0.77 (0.58–1.03)	1.22 (0.65–2.30)	3.22 (1.51–6.87)***
Middle	1.12 (0.82–1.53)	2.11 (1.24–3.60)**	0.81 (0.63–1.04)	1.29 (0.62–2.66)	0.72 (0.32–1.63)
High	reference	reference	reference	reference	reference

Abbreviation: SC, social capital.

*** *p* < 0.001

** *p* < 0.01

* *p* < 0.05.

Crude odds ratios (CORs) for SC dimensions varied across countries. In China, individual structural SC (COR = 2.42; 95% CI: 1.90–3.09) and community structural SC (COR = 1.74; 95% CI: 1.21–2.51) were significantly associated with edentulism. In South Africa, both individual and community structural SC were relevant. In contrast, Mexico and India showed no significant associations across SC dimensions. Ghana exhibited a significant association only with middle community structural SC (COR = 2.11; 95% CI: 1.24–3.60).

Multilevel regression results (Table 4) are presented for China, South Africa, and Ghana, where SC components showed significant associations. Countries with no significant associations (Mexico and India) are detailed separately in Table 5.

**Table 4:** Odds ratio and 95% confidence interval for associations between social capital and edentulism by multilevel logistic regression models.

	China					Ghana					South Africa				
Fixed effect	M0	M1	M2	M3	M4	M0	M1	M2	M3	M4	M0	M1	M2	M3	M4
Individual cognitive SC															
Low			1.27 (0.97–1.66)	1.29 (0.97–1.70)	1.30 (0.97–1.74)			1.02 (0.51–2.04)	1.02 (0.53–1.96)	1.05 (0.59–1.86)			0.52 (0.26–1.03)	0.52 (0.25–1.07)	0.48* (0.24–0.98)
Middle			1.06 (0.81–1.39)	1.09 (0.84–1.42)	1.08 (0.82–1.43)			0.88 (0.46–1.69)	0.94 (0.49–1.81)	0.98 (0.53–1.83)			0.64 (0.34–1.19)	0.79 (0.44–1.42)	0.80 (0.40–1.55)
High			ref	ref	ref			ref	ref	ref			ref	ref	ref
Individual structural SC															
Low			2.34*** (1.82–3.00)	1.58**(1.21–2.05)	1.43* (1.08–1.89)			1.77* (1.01–3.11)	1.28 (0.68–2.39)	1.25 (0.66–2.37)			2.54** (1.33–4.86)	2.62**(1.35–5.08)	2.34** (1.16–4.74)
Middle			1.46*** (1.18–1.80)	1.24 (0.99–1.55)	1.18 (0.93–1.50)			1.67 (0.83–3.37)	1.47 (0.69–3.12)	1.55 (0.71–3.39)			1.31 (0.68–2.52)	0.96 (0.46–2.00)	0.87 (0.39–1.92)
High			ref	ref	ref			ref	ref	ref			ref	ref	ref
Community cognitive SC															
Low		0.91 (0.63–1.31)	0.83 (0.58–1.20)	0.83 (0.57–1.20)	0.79 (0.54–1.15)		0.91 (0.53–1.57)	0.86 (0.47–1.59)	0.63 (0.34–1.18)	0.59 (0.33–1.08)		0.54 (0.27–1.07)	0.68 (0.32–1.45)	0.93 (0.41–2.10)	1.09 (0.47–2.53)
Middle		1.04 (0.72–1.51)	0.98 (0.68–1.42)	0.85 (0.58–1.25)	0.83 (0.57–1.21)		1.12 (0.63–2.00)	1.40 (0.81–2.42)	1.31 (0.76–2.23)	1.23 (0.73–2.08)		0.65 (0.29–1.47)	0.75 (0.32–1.78)	0.84 (0.34–2.05)	0.98 (0.40–2.42)
High		ref	ref	ref	ref		ref	ref	ref	ref		ref	ref	ref	ref
Community structural SC															
Low		1.76** (1.22–2.53)	1.32 (0.90–1.93)	2.10*** (1.42–3.10)	1.95*** (1.32–2.90)		1.12 (0.63–2.00)	0.97 (0.54–1.75)	1.04 (0.58–1.88)	1.03 (0.57–1.88)		3.4**(1.58–7.32)	2.45*(1.12–5.37)	1.81 (0.79–4.13)	1.85 (0.81–4.24)
Middle		1.15 (0.82–1.60)	0.98 (0.68–1.42)	1.27 (0.87–1.85)	1.35 (0.93–1.96)		2.06** (1.22–3.47)	1.89** (1.11–3.22)	1.78* (1.04–3.00)	1.76* (1.05–2.96)		0.74 (0.34–1.62)	0.62 (0.28–1.36)	0.50 (0.22–1.17)	0.44 (0.18–1.05)
High		ref	ref	ref	ref		ref	ref	ref	ref		ref	ref	ref	ref
Random effect															
Community variance	0.51 (0.09)	0.46 (0.09)	0.47 (0.10)	0.41 (0.08)	0.38 (0.09)	0.82 (0.22)	0.57 (0.17)	0.58 (0.18)	0.33 (0.16)	0.28 (0.16)	5.92 (1.13)	4.98 (0.98)	4.85 (0.99)	3.81 (0.93)	3.93 (1.05)
ICC (%)	13	12	13	11	10	20	14	14	9	7	64	60	59	54	54

*Note:* Estimates for Mexico and India are not shown because the univariable association between social capital and edentulism was not statistically significant. M0: null model without explanatory variables. M1: model with community‐level social capital. M2: model with individual and community‐level social capital. M3: adjusted for sociodemographic variables. M4: adjusted for sociodemographic variables, behavioral risk factors, and chronic diseases

Abbreviations: ICC, intraclass correlation; ref, reference; SC, social capital.

*** *p* < 0.001

** *p* < 0.01

* *p* < 0.05.

**Table 5:** Odds ratio and 95% confidence interval for associations between social capital and edentulism by multilevel logistic regression models (India and Mexico).

Fixed effect	India					Mexico				
	M0	M1	M2	M3	M4	M0	M1	M2	M3	M4
Individual cognitive SC										
Low			1.30 (0.90–1.87)	1.20 (0.86–1.68)	1.19 (0.85–1.67)			0.85 (0.38–1.87)	0.84 (0.40–1.77)	0.81 (0.38–1.71)
Middle			1.45 (0.87–2.41)	1.33 (0.87–2.03)	1.34 (0.87–2.06)			0.75 (0.35–1.57)	0.72 (0.36–1.45)	0.72 (0.37–1.42)
High			reference	reference	reference			reference	reference	reference
Individual structural SC										
Low			1.26 (0.87–1.83)	0.83 (0.59–1.16)	0.83 (0.58–1.17)			1.03 (0.49–2.17)	0.70 (0.31–1.55)	0.84 (0.41–1.72)
Middle			1.18 (0.89–1.57)	1.02 (0.74–1.42)	1.03 (0.74–1.43)			1.97 (0.64–6.04)	1.17 (0.54–2.53)	1.15 (0.54–2.44)
High			reference	reference	reference			reference	reference	reference
Community cognitive SC										
Low		0.98 (0.74–1.29)	0.89 (0.63–1.26)	0.88 (0.62–1.25)	0.86 (0.61–1.23)		0.52 (0.24–1.16)	0.54 (0.21–1.39)	0.71 (0.30–1.72)	0.73 (0.30–1.79)
Middle		0.90 (0.69–1.18)	0.85 (0.63–1.15)	0.90 (0.66–1.24)	0.89 (0.65–1.23)		0.63 (0.29–1.39)	0.62 (0.26–1.46)	0.64 (0.29–1.40)	0.58 (0.27–1.27)
High		reference	reference	reference	reference		reference	reference	reference	reference
Community structural SC										
Low		0.77 (0.58–1.03)	0.72 (0.51–1.02)	0.78 (0.54–1.13)	0.78 (0.55–1.11)		1.43 (0.68–3.00)	1.37 (0.63–3.00)	1.29 (0.60–2.77)	1.17 (0.56–2.45)
Middle		0.82 (0.64–1.05)	0.79 (0.61–1.03)	0.83 (0.62–1.10)	0.83 (0.63–1.10)		1.45 (0.69–3.01)	1.40 (0.67–2.95)	1.66 (0.82–3.36)	1.59 (0.79–3.21)
High		reference	reference	reference	reference		reference	reference	reference	reference
Random effect										
Community variance	0.49 (0.09)	0.48 (0.09)	0.48 (0.09)	0.56 (0.10)	0.54 (0.10)	1.83 (0.57)	1.80 (0.57)	2.04 (0.69)	1.58 (0.66)	1.51 (0.64)
ICC (%)	13.1	12.6	12.7	14.5	14.0	35.0	35.0	38.0	32.0	31.5

*Note:* M0: null model without explanatory variables. M1: model with community‐level social capital. M2: model with individual and community‐level social capital. M3: adjusted for sociodemographic variables. M4: adjusted for sociodemographic variables, behavioral risk factors, and chronic diseases

Abbreviations: ICC, intraclass correlation; SC, social capital.

Model 0 confirmed community‐level variance, justifying multilevel modeling. In Model 1, low community structural SC was associated with higher edentulism odds in China and South Africa, and moderate levels in Ghana. Model 2 added individual SC components, with structural SC emerging as significant in China and South Africa. In Ghana, both low individual structural SC and moderate community structural SC were associated with edentulism. Model 3 controlled for sociodemographic factors. Structural SC at both the individual and community levels remained significantly associated with edentulism in China. In South Africa, individual structural SC remained significant. In Ghana, the association persisted for the middle community structural SC only. Model 4 incorporated all variables, including health behaviors and chronic conditions. In China, individual and community structural SC retained their associations with edentulism (OR = 1.43; 95% CI: 1.08–1.89 and OR = 1.95; 95% CI: 1.32–2.90, respectively). In South Africa, low individual structural SC increased edentulism odds (OR = 2.34; 95% CI: 1.16–4.74), while low cognitive SC showed a protective association (OR = 0.48; 95% CI: 0.24–0.98). In Ghana, middle community structural SC remained significantly associated with edentulism (OR = 1.76; 95% CI: 1.05–2.96). No significant associations were observed in India or Mexico across all models.

A forest plot (Figure 2) visually summarizes the multilevel regression findings (Model 4) across countries. ICCs decreased from Model 0 to Model 4 in China, South Africa, and Ghana, indicating improved model fit.

In addition, formal statistical comparisons of effect sizes between countries were not conducted due to methodological differences and sample heterogeneity; the comparisons are descriptive in nature, which is acknowledged as a limitation and an area for future research.

## 4 Discussion

This study used a multilevel analysis to explore the association between SC components and self‐reported oral health (edentulism) in diverse contexts while accounting for several covariates (sociodemographic factors, health behaviors, and chronic diseases). The findings revealed variations in the relationship between SC dimensions and edentulism across the studied countries.

In China, our study found that edentulism was independently associated with low structural SC and those living in neighborhoods with low structural SC after adjusting for confounding factors. Additionally, the cognitive SC dimension was not statistically significant in predicting edentulism. Among Chinese adults, structural SC exerts a stronger influence than cognitive SC on edentulism. These findings are consistent with those reported by Li et al. (2022). Earlier studies have also demonstrated the association between social participation, a key component of structural SC, and edentulism. In a southeastern Brazilian city, a cross‐sectional study involving 163 individuals aged 60 and older showed an association between social participation and edentulism among older Brazilian adults, determined through questionnaires and oral examinations (Rodrigues et al. 2012). In Japan, a cross‐sectional study among older individuals suggests that social participation helps maintain oral health. Participation in sports groups, neighborhood community associations, or hobby clubs could be a strong predictor of maintaining teeth in later years (Takeuchi et al. 2013). Furthermore, good social relationships correlate with improved self‐rated oral health and greater tooth count (Koyama et al. 2016; Aida et al. 2009; Tsakos et al. 2013).

The results in South Africa and Ghana differed from those in China. In South Africa, individual‐level cognitive and structural SC were associated with edentulism, while community SC dimensions were not. At the same time, contextual‐level structural SC had a more significant effect on oral health outcomes than individual‐level structural SC in Ghana. The outcomes in South Africa align with the conclusions drawn by Nogueira et al. (2019), who found an association between poor SC, tooth loss, and dental caries in Brazilian adults. However, their investigation solely focused on individual SC dimensions, employing levels of collaboration, safety, and happiness as measurement tools. In contrast, our study used trust, safety, and involvement in formal and informal social activities as indicators of individual SC.

To date, evidence suggests that there are stronger associations between health and trust (cognitive SC) than between health and associational membership (structural SC) (Kim et al. 2008). Harpham suggests measuring SC based on cognitive aspects, which relate to how people feel (e.g., trust, values), and structural aspects, which relate to what people do (e.g., participation, associational links), as the two types of factors are likely to influence health through different mechanisms (Kawachi et al. 2008). Our study did not show a significant association between cognitive SC and edentulism except in South Africa; this finding may suggest contextual or cultural influences in how individuals perceive and respond to trust‐based social environments. However, given the cross‐sectional design, this interpretation remains hypothesis‐driven and should be further explored in future studies.

In addition, a literature review by Kim et al. indicated stronger individual‐level associations than community‐level associations for the same indicator, emphasizing that personal social connections have a greater impact on health and well‐being than broader community‐level factors (Kim et al. 2008). However, as mentioned earlier, Ghana had different results; the community structural SC was stronger than the individual level. Our findings align with Chola and Alaba, who explored the association between SC and self‐rated health across nine South African provinces. Their findings revealed health variations across different provinces, highlighting the importance of accounting for contextual factors, such as regional disparities in healthcare access, cultural norms, economic conditions, and the strength of community networks, when analyzing the association between SC and health, even within a single country (Chola and Alaba 2013).

In Mexico and India, despite covariate control, none of the aspects of SC were associated with edentulism in our study. This absence of significant findings warrants further reflection. Low response rates (53% in Mexico and 68% in India) could account for this lack of significance, as both countries had the lowest rates compared with others. Such rates may introduce nonresponse bias, affecting variables' association and potentially skewing results. Additionally, given the potential differences in these countries' characteristics, another explanation might be the need for a more country‐specific SC measurement tool. Alternatively, strong family‐based networks—common in both India and Mexico—may fulfill social roles typically attributed to broader community capital, reducing the measurable impact of structural or cognitive SC on oral health outcomes.

Furthermore, India is a land of diverse religions and cultures; it is essential to understand how people of various societies, religions, and classes organize themselves to form SC (Himanshu et al. 2019). Indian and Mexican families may vary by region, class, and urban or rural areas, but they generally maintain strong family connections; three or four generations living together is not uncommon. In contrast, China implemented its “one‐child policy” for an extended period, increasing the number of elderly people living alone (Li et al. 2022). The presence of children and having a larger number of children increases the likelihood of rotational living, shared responsibilities, and caregiving for elderly parents. Older individuals benefit from social and familial support (bonding SC), experiencing greater feelings of love, enhanced confidence in dealing with health issues, and improved self‐esteem and overall well‐being. These factors can potentially influence the outcome variable (self‐reporting edentulism) in the current study. Additionally, individuals with strong family ties may not perceive the need for community mobilization or social participation beyond their immediate family connections. Therefore, different societies exhibit diverse sociocultural norms, variations in family structures, and distinct economic standings. The pace of urbanization, modernization, and their impacts on family dynamics, health, and well‐being among older adults can differ across countries.

Research on the association between oral health and SC is more limited relative to general health. A few studies have examined the potential impact of SC on oral health, particularly in LMICs (Batra et al. 2014; Knorst et al. 2022). Cultural and historical variations in Western countries raise questions about the applicability of previous findings to the association between SC and oral health in LMICs. Moreover, disparities in indicators, SC measures, and analytical approaches hinder direct study comparisons. The absence of validated standardized questionnaires in the literature also impedes global comparisons (Knorst et al. 2022).

This study highlights the role of chronic diseases, formal education, and health behaviors in increasing the odds of edentulism, consistent with previous research (Kailembo et al. 2016). Chronic diseases were linked to edentulism across all five countries, while the lack of formal education in India and health behaviors, such as smoking and poor diet, were associated with higher odds of edentulism, particularly in China and South Africa. These factors may act as mediators, influencing the relationship between SC and edentulism. Further research is needed to explore their mediating role in understanding how SC impacts oral health.

The mechanisms by which SC affects oral health are not yet fully understood, and different explanations have been proposed; high levels of SC have been shown to affect health‐related behaviors via social support, informal social control, collective action, and sharing information among others in a social network (Carpiano 2007). Lower levels of SC have also been related to psychological distress, which can lead to engagement in unhealthy habits, such as smoking, alcohol use, and eating unhealthy food; these unhealthy behaviors can increase dental caries, periodontal diseases, and tooth loss (Rouxel et al. 2015).

Active aging is influenced by transportation, a challenge in many LMICs due to inadequate infrastructure, poor roads, limited public transit, and rural isolation, impacting access to vital services such as healthcare (Patil et al. 2022). SC can enhance dental care access through network support for attending appointments and transportation; studies have shown that people who live alone or unmarried use dental services less often than others (Wu et al. 2005; Burr and Lee 2013). Additionally, community‐level SC facilitates collective action, including advocating for better dental care by engaging authorities, potentially yielding positive improvements in dental healthcare. Moreover, community SC enhances health via social contagion. Trusting social networks leads to adopting positive health norms and healthy behaviors. SC can spread oral health knowledge, promote good habits, and deter unhealthy actions through social influence and norms (Rouxel et al. 2015).

These findings have potential policy and clinical implications, especially for LMICs. Strengthening social networks at both the community and household levels—through targeted community engagement, oral health promotion programs, and supportive infrastructure—may reduce oral health disparities. Policymakers should consider integrating SC components into primary oral healthcare planning and delivery, particularly in underserved areas.

SAGE Wave 1 relied solely on self‐reported data without objectively validating edentulism diagnoses, potentially resulting in underestimated prevalence rates. The data set also lacked important information on denture use and oral hygiene practices, which could have influenced the findings. Previous research indicated disparities between clinical and subjective oral health measurements. Clinical indicators assess oral pathologies, such as dental caries and periodontal diseases, while self‐reported markers encompass subjective feelings of dental health and overall well‐being. Nonetheless, large‐scale population surveys commonly employ self‐reported methods, which are consistently validated for accurate health event reporting (Wu et al. 2015).

### 4.1 Methodological Considerations

A key strength of our study is the inclusion of SC at various levels in our analysis, consistent with the consensus among researchers that both individual and collective SC are essential for understanding its impact (Harpham 2008). However, given the absence of temporal sequencing information between SC and edentulism, establishing causality is not possible. Reverse causality may play a bidirectional role in the association between SC and edentulism. On one hand, individuals with low SC, characterized by limited trust, weak networks, or minimal social participation, may be less likely to access preventive dental care, health information, or community resources, which can increase their risk of tooth loss. On the other hand, edentulism itself may lead to reduced social interaction due to feelings of embarrassment, diminished self‐esteem, or difficulty eating and speaking. This can result in social withdrawal and declining engagement with community or social groups, thereby lowering both perceived and actual SC. These mutually reinforcing pathways suggest a complex, dynamic relationship between oral health and SC that cannot be fully understood through cross‐sectional data alone. Longitudinal research is therefore essential to disentangle the temporal sequence and explore how these factors evolve over time.

Additionally, as the interviewer administered the questionnaire, there was a potential for participants to provide inaccurate responses, particularly in relation to questions about smoking and alcohol consumption. Nonetheless, the anonymity maintained during survey completion likely mitigated intentional misreporting.

The present study has illuminated the association between SC and oral health across diverse contexts. However, it is important to note that edentulism was assessed using a single question, which may not capture the full complexity of the relationship. Clinical edentulism measurements could reveal different dynamics between SC and oral health. Furthermore, the study's reliance on secondary data and its specific design limitations have hindered the exploration of key factors, such as oral hygiene practices, cross‐cultural perceptions of edentulism, dental service utilization, and the direct measurement of community‐level SC. Addressing these aspects could provide valuable insights for future research. In addition, future research should analyze country‐specific cultural norms impacting SC and oral health. Future studies should also explore how cultural norms, gender roles, and familial structures shape the relationship between SC and oral health. Mixed‐method approaches may be especially useful in unpacking subjective experiences and contextual nuances.

## 5 Conclusion

Our study evaluated the association of both cognitive and structural components of SC and edentulism at the individual and community levels in the five LMICs. The findings revealed diverse country‐specific associations and contextual variations in how SC influences oral health. The complexity of SC was evident; this study offers a foundation for future research, illuminating the role of SC in older adults' edentulism across diverse contexts. Longitudinal studies are needed for deeper insight into SC's impact on oral health. Programs enhancing oral health through SC in adults should consider country‐specific (e.g., China's “one‐child policy”) and sociocultural factors (e.g., community social cohesion).

## Author Contributions

Saida Ben‐Bihi, Yanfei Guo, and Junmei Miao Jonasson conceived the study. Saida Ben‐Bihi conducted the data analysis and drafted the manuscript, with Yanfei Guo providing guidance on statistical methods and strategies. Yanfei Guo and Junmei Miao Jonasson reviewed the data analysis. Yanfei Guo, Junmei Miao Jonasson, Richard Olofsson, and Hülya Çevik‐Aras contributed to editing, revising, and finalizing the manuscript. All authors thoroughly reviewed and approved the final version.

## Ethics Statement

Our data were derived directly from publicly accessible Global Ageing and Adult Health (SAGE) data containing no individually identifying information about survey participants. The WHO Ethical Review Committee reviewed and approved the SAGE survey (RPC149). Implementing partner institutions in each country provided ethical approval for SAGE, ensuring adherence to all ethical considerations, including written informed consent. Hence, no ethical approval or informed consent was needed for the present study.

## Conflicts of Interest

The authors declare no conflicts of interest.

## Data Availability

The SAGE data are publicly available and can be accessed for research purposes with prior permission from http://apps.who.int/healthinfo/systems/surveydata/index.php/catalog/sage.
